# Epigenetics and Neurological Disorders in ART

**DOI:** 10.3390/ijms20174169

**Published:** 2019-08-26

**Authors:** Marina La Rovere, Marica Franzago, Liborio Stuppia

**Affiliations:** 1 Department of Psychological, Health and Territorial Sciences, School of Medicine and Health Sciences, “G. d’Annunzio” University, 66100 Chieti-Pescara, Italy; 2 Department of Medicine and Aging, School of Medicine and Health Sciences, “G. d’Annunzio” University, 66100 Chieti-Pescara, Italy; 3 Aging Center Studies-Translational Medicine (CeSI-Met), “G. d’Annunzio” University, 66100 Chieti-Pescara, Italy

**Keywords:** neurological disorders, epigenetics, ART

## Abstract

About 1–4% of children are currently generated by Assisted Reproductive Technologies (ART) in developed countries. These babies show only a slightly increased risk of neonatal malformations. However, follow-up studies have suggested a higher susceptibility to multifactorial, adult onset disorders like obesity, diabetes and cardiovascular diseases in ART offspring. It has been suggested that these conditions could be the consequence of epigenetic, alterations, due to artificial manipulations of gametes and embryos potentially able to alter epigenetic stability during zygote reprogramming. In the last years, epigenetic alterations have been invoked as a possible cause of increased risk of neurological disorders, but at present the link between epigenetic modifications and long-term effects in terms of neurological diseases in ART children remains unclear, due to the short follow up limiting retrospective studies. In this review, we summarize the current knowledge about neurological disorders promoted by epigenetics alterations in ART. Based on data currently available, it is possible to conclude that little, if any, evidence of an increased risk of neurological disorders in ART conceived children is provided. Most important, the large majority of reports appears to be limited to epidemiological studies, not providing any experimental evidence about epigenetic modifications responsible for an increased risk.

## 1. Introduction

Almost 40 years as have passed since the first report of a child born by the use of In Vitro Fertilization (IVF) [1] and more than 25 years from the first application of the Intracytoplasmic Sperm Injection (ICSI) [2]. These two major breakthroughs, representing crucial steps in the field of Assisted Reproduction Techniques (ART), generated a new era for the human reproduction. In fact, since their first pioneering applications, an increasing number of couples attend ART protocols to generate a baby and it has been estimated that more than 200,000 infants are annually born worldwide by this technology, for a total of over 5 million children, accounting for 1% of births in the US and 4.3% of births in Europe and Australia [3].

Several concerns about the safety of ART had been initially raised by different authors, who suggested an increased risk of malformations in the offspring generated by in vitro fertilization [4,5]. However, after a more careful analysis of epidemiological data, it has been verified that ART is associated with a small increase in birth defect, being the anomaly rate at birth 3–4% versus 2–3% in natural reproduction [6,7]. Anyway, this slightly increased risk does not appear to discourage couples to undergo ART when this approach represents the only possibility to generate a child.

More recently, novel attention has been devoted to the health risk in ART offspring due to the evidence of an increased prevalence of imprinting disorders, such as Beckwith–Wiedemann syndrome (BWS) [8]. In particular, it was evidenced that BWS patients born after ART more frequently showed epigenetic than genetic alterations as compared to BWS children born by natural fertilization [9]. Subsequently, an increasing amount of data reinforced the hypothesis of an epigenetic disturbance induced by ART, related both to the poor quality of gametes and to the technical procedures used during in vitro fertilization [10].

These observations lead to crucial questions concerning the safety of ART, namely: Are children generated by IVF actually at risk of late onset rather than at birth diseases? Does this risk involve also neurological disorders? Which mechanisms are involved?

To give an answer to these questions, here we report a detailed analysis of literature reports about ART and risk of late onset, mostly neurological, diseases, with particular attention to the epigenetic mechanisms involved in these risks. In particular, this review will be mainly focused on the effects of alterations in DNA methylation, since the majority of literature is centered on this kind of epigenetic modification.

## 2. Risk of Late Onset Diseases in ART Conceived Children

Evidence for an increased susceptibility to late onset diseases in the offspring generated by ART has been provided both by animal models and in human [11,12]. In animals generated by ART, several studies demonstrated a statistically significant increase in the prevalence of different adult onset disorders as compared to those generated by natural fertilization. In details, Calle et al. (2012) evidenced reduced fertility in mice produced by in vitro culture, which in turn transmitted organomegaly and glucose intolerance to their male offspring [13]. Cardiovascular dysfunction with shortened life span was evidenced by Rexhaj et al. (2013), who detected higher arterial blood pressure due to impaired endothelial-dependent artery vasodilation [14]. Donjacour et al. (2014) showed sex-related alterations in glucose metabolism and systolic blood pressure in male mice produced after IVF and suboptimal embryo culture conditions [15].

Significant cardiovascular changes have been subsequently detected also in human newborn generated by ART, with higher systemic blood pressure levels in IVF and ICSI children as compared to those spontaneously conceived, even after correction for birth weight, gestational age and body size [16,17,18]. Further evidence came from the studies of Liu et al. (2015), demonstrating significant changes in cardiac systolic and diastolic function during childhood in ART generated children, thus suggesting an increased risk of early onset myocardial alterations in this population [19]. Kosteria et al. (2017) investigated the proteomic profile of ICSI children, evidencing the presence of abnormal expression of proteins mostly involved in acute phase reaction, blood coagulation, complement pathway activation and iron and lipid metabolism [20]. Finally, in a systematic review and meta-analysis, Xiao-Yan et al. (2017) demonstrated the presence in ART children of a minor yet statistically significant increase in blood pressure, a suboptimal cardiac diastolic function, and a higher vessel thickness, suggesting an increased risk of cardiovascular disease [21].

Although cardiovascular function appears to represent the main condition affected by ART, to the availability of a longer follow up have evidenced other adult onset dysfunctions. In fact, Belva et al. (2016) described a reduced sperm concentration in ICSI children as compared to boys spontaneously conceived, as well as a two-fold lower total sperm count and total motile count [22]. On the other end, inconsistent results have been showed by studies investigating the risk of neurological defects in ART conceived children. This topic will be specifically treated in the paragraph “Are ART conceived children at increased risk of neurological disorders?”.

## 3. Epigenetic Alterations in ART

The above described effects of ART on the long-life health of children are likely related to epigenetic mechanisms. In fact, early studies on animal models evidenced that ovulation induction, eggs manipulation and embryo culture are all able to induce altered DNA methylation in the offspring generated by ART [23,24,25,26,27].

Recent studies have confirmed the majority of data obtained in animal models also in human.

Choux et al. (2018) recently demonstrated that DNA methylation of imprinted loci and transposable elements (TE) were significantly lower in ART placentas than in control placentas [28]. Even though all of the newborns investigated in this study were healthy, authors raised the question about of the potential long-term effects of these epigenetic modifications [28].

Among the possible causes of epigenetic modifications in ART, superovulation has been invoked as a possible factor. In fact, Velker et al. (2017) observed that superovulation with low and high hormone treatment resulted in disruption of imprinted methylation at the maternal *Mest* allele in blastocysts [29]. In addition, related to cultured embryos during preimplantation mouse development, both the Fast and Slow culture groups experienced a significant loss of maternal *Mest* methylation compared to in vivo-derived controls [29]. These results suggested that *Mest* gDMR methylation was less stable in ART-produced pre implantation embryos than other imprinted gDMRs.

In addition to the epigenetic alterations, induced on female gamete by ovarian stimulation and eggs manipulation, a further effect of culture conditions in ART is represented by epigenetic modifications induced in the embryo during the progression from conception through first cleavage to morula and blastocyst stages. In natural conception, this progression occurs in the oviductal fluid making the embryo highly sensitive to several maternal conditions (diet, presence of metabolic and inflammatory dysfunctions) [10,30], while in IVF, it occurs “in vitro”, being thus susceptible to variations in cell culture medium composition, O_2_, pH and temperature [31].

Also, the process of embryo transfer can induce loss of methylation on the maternal allele of the KvDMR1 locus (one of the possible causes of BWS in human) [32]. In this view, the design of new devices for the embryo transfer, able to avoid the exposure of the embryos to even subtle variations in their environmental conditions has been suggested [33].

Besides culture conditions, also the quality of the gametes used in ART can represent a risk factor for epigenetic defects, since it has been demonstrated that sperm of oligozoospermic man can show the presence of DNA methylation changes at imprinted loci [34,35,36]. In this view, it has been recently suggested that a careful assessment of spermatozoal parameters is essential to achieve embryo development [37].

Choufani et al. (2018) using genome-wide profiling, examined the extent of epigenetic abnormalities in matched placentas from an ART/infertility group and control singleton pregnancies from a prospective longitudinal birth cohort, suggesting the importance of considering both sex and paternal factors [38]. Placentas from pregnancies conceived with IVF/ ICSI showed distinct epigenetic profiles as compared to those conceived with ovulation induction or intrauterine insemination [38]. Moreover, the authors observed a sub-group, enriched for paternal infertility and older paternal age, amongst the IVF/ICSI placentas, suggesting an interaction of infertility and techniques in perturbing the placental epigenome [38].

Another interesting topic is the one considering the relationship between parental constitutional genetic variants and epigenetic alterations after ART. In fact, Marjonen et al. (2018) recently evidenced that the presence of the rs10732516 polymorphism within the *H19* imprinting control regions (ICR) is able to affect the effects of ART in a parent-of-origin manner through DNA methylation changes in placental tissue [39].

A possible application of the increased knowledge about embryo DNA methylation and ART has been suggested by Li et al., (2017) who evidenced a direct relation of global methylation and embryo quality, suggesting DNA methylome as a potential biomarker in blastocyst selection in ART [40].

Since epigenetic alterations have been suggested to play a possible role in the increased risk of long-life diseases in ART children described in the previous section, their involvement in the susceptibility of other conditions is currently under investigation. In particular, great attention has been devoted to the risk of neurological disorders, since recent studies have evidenced that some of these conditions can be related to epigenetic dysfunctions.

## 4. Epigenetic Bases of Neurological Disorders

The first evidence of an involvement of epigenetic mechanisms in brain development, behavior and neurological disorders came from the studies on genomic imprinting (monoallelic parental depended gene expression), which, although involving a small portion of the human genome, plays a crucial role in the control of placental function and brain development. It must be stressed that genomic imprinting represents a very peculiar kind of epigenetic control of gene expression. In fact, the parental-specific expression of imprinted genes is regulated by allele-specific epigenetic marks which are established during gametogenesis and maintained throughout life. Imprinted genes reside in clusters throughout the genome, regulated by imprinting control regions (ICRs) that are methylated in a sex-specific manner during gametogenesis. This methylation is maintained after fertilization when most of the genome is being reprogrammed [41]. More than 300 imprinted genes are expressed in mouse brain, two third of which involving 26 brain regions [42,43].

Several imprinted genes (such as *Peg3*, *Peg1*, *Zack1* and *Nnat*) are co-expressed in hypothalamus and placenta [43], with the placental genome directly affecting maternal hypothalamic function in order to stimulate post-partum maternal care and milk letdown [44]. Since in the same period the hypothalamus of the fetus is developing as well, this mechanism allows selection pressure to operate across two generations. [45]. In human, alterations of the genomic imprinting are responsible for a small group of syndromes such as, Prader–Willi syndrome (PWS) and Angelman syndrome (AS) [46]. PWS is a neuroendocrine and behavioral disorder due to loss of function of the paternally imprinted genes in the 15q11–q13 region, characterized by hypotonia and feeding difficulties after birth, replaced after the first months by hyperphagia and undiscerning eating, with development of morbid obesity. PWS patients also display hypogonadism, cognitive delays, low levels of testosterone, gonadotropins, GH and IGF1 [47,48].

Developmental delay, microcephaly, intellectual disability with absent or limited speech, gait ataxia and behavioral profile with happy demeanor are the typical signs of AS [49], caused by loss of maternal expression of *UBE3A*.

In addition to the genomic imprinting, other epigenetic factors play key roles in promoting cellular development, plasticity, differentiation and stress responses within the nervous system [50], and epigenetic processes are now often invoked as involved in the pathogenesis of a number of nervous system diseases [51]. One of the most important example is provided by the model of the Rett Syndrome, a progressive neurodevelopmental disease affecting females, characterized by repetitive and stereotypic hand movements replacing purposeful hand use, gait ataxia, seizures and autistic features [52]. This disease is caused by an X-linked gene encoding *MeCP2*, whose function is to bind methylated DNA and to repress transcription in a methyl-CpG-dependent manner.

Other crucial genes involved in the regulation of cerebral homeostasis can be affected in their function by epigenetic alterations. Among these, great attention has been devoted to the *BDNF* gene, a member of the family of neurotrophine proteins, that plays a fundamental role in the development, maintenance and plasticity of the central nervous system [53]. *BDNF* performs its function both in prenatal age and in adult neurogenesis and plays a critical role in learning, memory and cognition in the cerebral cortex and the hippocampus. Several studies reported a deregulation of the expression of BDNF by alterations of DNA methylation and non-coding RNA in different neurological diseases, such as Alzheimer’s disease (AD), Parkinson’s disease (PD), Huntington’s disease (HD) and amyotrophic lateral sclerosis (ALS) [54,55]. More recently, *BDNF* dysregulation has been described also mediated by histone modifications [56].

Epigenetic dysfunctions in AD have been identified in vitro, in animals models and in human patients [57,58,59]. Mastroeni et al. (2010) showed a clear decrease in DNA methylation markers in cortical neurons in AD patients as compared to normal elderly controls, but this variation was not found in the cerebellum, a region that is generally not involved in the disease [60].

Regarding PD, early studies had been focused on the analysis of the promoter methylation levels of genes responsible for the monogenic, early onset, rare forms of PD, by analyzing DNA methylation in post-mortem brain tissue and peripheral blood, with inconsistent results [61]. However, more recent studies have investigated the epigenetic structure of the *SNCA* gene, encoding the α-synuclein protein, considered as an important component of Lewy’s bodies, a typical neuropathological trait of PD patients. DNA methylation of this gene may be involved in the pathogenesis of the disease through structural changes or by overexpression of the protein, with consequent accumulation and protein aggregation [62]. Moreover, Guhathakurta et al. (2017) discovered that this gene contains several transcriptionally activate histone modifications and associated potential transcription factor binding sites in the non-coding areas, that strongly suggests alternative mechanisms of regulation pathways [63].

Another field of interest is represented by the relationship between epigenetic modifications and intellectual disability (ID) Currently, more than 500 genes have been identified as involved in ID etiology, often affecting the same metabolic pathway [64]. Several of these genes encodes for epigenetic regulators such as chromatin factors, which are directly involved in the regulation of chromatin structure at level of genes fundamental in neurodevelopment [65]. For example, some of these belong to DEAD/H ATPase family, which has a key role in positioning of nucleosomes. This family of proteins can be further divided into four subfamilies, SWI/SNF, INO80/SWR1, ISWI, and CHD ATPases [66]. Mutations in genes encoding SWI/SNF family proteins have been associated in Coffin–Siris syndrome, which is characterized by variable phenotypic manifestations including ID [67].

The influence of epigenetic modifications has also been reported in other neurological disorders, such as epilepsy. In fact, convulsions in epileptic subjects can generate epigenetic changes in the brain in gene expression patterns, contributing in turn to the distinctive features of epilepsy [68]. Some studies have shown that the induction of convulsions in animal models lead to overexpression of REST/NRSF both at mRNA and protein levels [69,70]. This protein acts as a transcription repressor of a large subset of genes during neurogenesis, suggesting that convulsion can cause an imbalance in the epigenetic mechanisms that control important processes in brain.

Another contributes to the knowledge about the epigenetic modifications in epileptic subjects came from a study by Kobow et al. (2013), who reported a global hypermethylation in chronic epileptic rats. This study demonstrated that in presence of a ketogenic diet there was a reduction in the frequency of seizures and a change in the DNA methylation levels, suggesting a close correlation between nutrition and epigenetic modifications [71].

Miller-Delaney et al. (2012), reported changes in methylation patterns both in epileptic animals and in those with epileptic tolerance. Epileptic tolerance is an endogenous protective mechanism in brain in response to a previous exposure to a non-damaging crisis before the epileptic status. Interestingly, an altered DNA methylation of several genes has been observed both in the presence of an endogenous tolerance and in the epileptic seizure without preconditioning. In fact, 321 genes showed more than 90% of their promoters hypomethylated. Among these, new genes never associated before with epilepsy, such as the polychrome *Phc2* gene were identified [72].

Particular attention has been devoted to the hypothesis that epigenetic mechanisms could be related to neurodevelopmental disorders, including Autism Spectrum Disorder (ASD).

Sun et al. (2016) focused on the signature of histone acetylation analyzing global acetylome in brain samples of post-mortem human ASD patients compared to normal control brain samples, evidencing a common acetylomic marks in more than 68% of the syndromic samples in more than 5000 cis-regulatory elements. Furthermore, there was a strong increase of gene expression related to ion channels, synaptic function and epilepsy/neuronal excitability, which had already been described as dysregulated in ASD [73].

Although these emerging data support a correlation between epigenetics and susceptibility to ASD, other studies affirm the opposite, highlighting how current knowledge is not sufficient yet to definitively clarify this association. In fact, Ginsberg et al. (2012) investigated the whole genome gene expression and DNA methylation by microarrays in post-mortem brain tissue between ASD patients and control subjects, reporting no significant changes in DNA methylation inside the two group [74]. Therefore, the authors concluded that the changes detected in gene expression in ASD may be the result of other regulatory mechanisms involved, other than methylation.

For this reason, further studies should be promoted to shed light on the mechanisms by which these epigenetic modifications are effectively implicated in neurological disorders and in particular how different environmental factors can mediate these changes.

## 5. Are ART Conceived Children at Increased Risk of Neurological Disorders?

As above described, a clear evidence exists that (i) ART protocols can induce epigenetic modifications affecting embryo development and long-life health of the offspring; (ii) epigenetic modifications can be involved in the pathogenesis of neurological disorders.

As a consequence, the next question is: Are children generated by ART at the increased risk of neurological disorders during their life?

Early results on animal models suggested the possibility of long-term, even transgenerational consequences of ART on neurodevelopment and behavior of adult mice [11] (Table 1). Ecker et al. (2004) explored behavioral consequences of embryo culture in 129S6SvEvTacC57BL6J F1 mouse model, evidencing small but significant specific behavioral alterations in the elevated zero maze and Morris water maze tasks in adults derived from cultured embryos, likely as a consequence of a hippocampus dysfunction [75].

In the same period, Fernandez-Gonzalez et al. (2004) evidenced that mice derived from embryos cultured with Foetal Calf Serum (FCS) showed specific behavioral alterations in anxiety and deficiencies in implicit memories. Interestingly, these embryos at the blastocyst stage showed lower expression of H19 mRNA, although the differences were non-significant [76]. This result appears to be in agreement with previous studies evidencing a significantly higher level of methylation in fetuses produced from the culture of embryos in the presence of FCS [27].

More recently, Wu et al (2014) investigated the possible effect of blastomere biopsy in mice development, evidencing poorer spatial learning ability, increased neuron degeneration and altered expression of proteins involved in neural degeneration or dysfunction in the brain in aged biopsied mice as compared to aged control mice. At molecular levels, these authors detected a genome-wide low methylation in the brains of adult biopsied mice, with most of the involved genes associated with neural disorders. Authors concluded that an abnormal neural development and function in mice generated after blastomere biopsy is present, and that an impaired epigenetic reprogramming during early embryo development may be invoked as the mechanism leading to the impairment of the nervous system in the biopsied mice [77].

These results on animal models prompted researchers to investigate the risk of neurological disorders in children conceived by ART. Different and often inconsistent results have been obtained based on the investigated neurological defect [78] (Table 2).

An increased risk of Cerebral Palsy (CP) in ART conceived children has been reported by different authors. A study by Stromberg et al. (2002) [79] carried out on children conceived after IVF and followed up to 12 years evidenced an increased risk of CP (OR: 3.7), a finding confirmed by Lidegaard et al. (2005) [80] evidencing an 80% increased risk of CP in IVF children. Subsequently, other studies, although confirming the increasing risk of CP in ART conceived children, evidenced that this association was less evident after adjustment for multiple pregnancies preterm delivery or gestational age [81,82]. On the other hand, Liang Zhu et al. (2010) evidenced that in their series children born after ART had an increased risk of CP even after adjustment for preterm birth and multiplicity [83]. By analyzing in details a number of possible factors which co-vary both with IVF and with CP and after adjustment for year of birth, maternal age, parity, and smoking, Källén et al. (2010) concluded that ART is associated only to a moderately increased risk for CP, likely as a consequence of an increased risk of neonatal morbidity associated with multiple births [84].

Based on these results, it has been suggested that the increased risk of CP which is partly due to multiple births, partly to the neonatal morbidity seen after IVF, with a possible role played also by parental subfertility. A very recent study by Goldsmith et al. (2018) confirm this hypothesis, by evidencing that, despite the two-fold increase of CP after ART, after stratification for gestational age and plurality a residual risk remains in singletons born very preterm [85].

These observations overall seem to rule out a possible epigenetic mechanism in the pathogenesis of CP in ART conceived children, also considering the very limited data about epigenetic analysis carried out in in these children. However, a very recent report of Mohandas et al. (2018), carried out by genome-wide analysis of DNA methylation in 15 monozygotic twin pairs who later became discordant for CP, identified 33 Differentially Methylated Probes (DMPs) associated with CP in genes involved in immune signaling pathways or previously linked to epileptic encephalopathy [86]. Authors suggested a potential role for immune dysfunction in CP. Nevertheless, this study did not analyze children conceived by ART, and the presence of DMPs associated with CP does not indicate for sure a possible epigenetic dysfunction in CP.

The possible risk of intellectual disability in children conceived by ART represents another issue addressed by several studies. The majority of the reported cases appear to be reassuring, showing a minimal effect of the ART procedure in comparison to birth weight, gestational age, socio-economic status and parental educational in increasing the risk of intellectual disability [87,88,89,90]. However, it has been stressed that many of the reported studies have methodological limitations and that very limited data are available on adolescents and young adults conceived by ART [90]. Thus, further studies on longer follow up are required to fill these gaps and confirm the lack of increased risk of intellectual disability in ART conceived children.

Finally, several studies have investigated the association between ART and increased risk of autism or autism spectrum disorders (ASD), again with different results.

An early Danish study by Maimburg et al. (2007) suggested a decreased risk for developing infantile autism in ART children, even after adjustment for known risk factors associated with assisted conception and infantile autism [91]. Subsequently, another Danish study evidenced a slightly increased risk of ASD in ART children, that on the other hand disappeared after adjustment for maternal age, educational level, parity, smoking, birth weight and multiplicity [92]. The lack of association between ART and autism was further confirmed by other studies [87,88,89,90,91,92,93,94], but more recently additional studies have reported different results, raising again the question about a possible increased risk of ASD in ART conceived children. Kissin et al. (2015), although not detecting an overall increased prevalence of ASD in ART conceived as compared to normally conceived children, found a higher incidence during the first 5 years of life in children generated by ICSI as compared with IVF [95]. These results could suggest that also the type of technique chosen for fertilization could lead to different effects on the neurological development of the fetus. Liu et al., (2017), in a meta-analysis carried out on 3 cohort studies and 8 case-control studies, suggested a higher risk of ASD (RR = 1.35, 95% CI: 1.09–1.68, *p* = 0.007) in children generated by ART [96]. These authors suggested the presence of epigenetic changes induced by hormone exposure, semen preparation, freezing of embryos and gametes, use of culture media, growth conditions for embryos, and delayed insemination. However, this study did not analyze at molecular levels the presence of epigenetic alterations in ASD children conceived by ART, and such an association remains only a hypothesis.

Catford et al. (2017) carried out a systematic review of health outcomes of ICSI-conceived offspring beyond the neonatal period compared to IVF-conceived offspring with reassuring results related to neurodevelopment during infancy and childhood; whilst, data on neurodevelopmental disorders, growth, physical health and childhood cancer were inconclusive or limited [97]. Then, Levin (2018) showed that neurologic morbidity was significantly more common in IVF (3.7%) and OI (4.1%) offspring (up to 18 years) as compared with those following spontaneous pregnancies (3.1%; *p* = 0.017) [98]. In addition, Davidovitch (2018) observed that IVF treatment compared with spontaneous conception was not significantly associated with the ASD risk, whereas progesterone hormone treatment was associated with an increased risk of ASD (RR = 1.51, 95% CI 1.22, 1.86) compared to the group with no progesterone treatment [99]. The authors suggested that this effect may possibly reflect epigenetic modifications [99].

Although accumulating data suggest that individuals conceived by ART may have an increased risk of chronic metabolic disorders, to date, the human studies are small and not conclusive [100,101,102]. The methodological discrepancies among studies is due to inclusion criteria of subjects, sample size, sampling of the comparison group, parental characteristics and also the ART technique employed [103]. The health implications of IVF are under-studied, therefore longitudinal follow-up is clearly warranted to understand the potential long-term effects in ART offspring [12].

## 6. Discussion

Currently, there is an ongoing debate as to whether techniques and processes such as controlled ovarian hyperstimulation, IVF, ICSI, embryo cryopreservation, pre-implantation genetic diagnosis (PGD) and preimplantation genetic testing for aneuploidy (PGT-A) could cause alteration of gamete, embryo and fetal developments [104]. Since the ARTs procedures require multiple manipulations to the gamete and embryo during critical windows of epigenetic reprograming, the association between ART and aberrant epigenetic modifications, mostly evidenced in animal models, is not surprising [105]. In particular, embryo biopsy and PGT-A could improve the rate of implantation and clinical pregnancy [106], especially for the older patient population, despite the concerns over the neurodegeneration and dysfunction in the offspring raised in the past by some studies [77,107,108]. For example, Wu et al. (2014) showed an abnormal neural development and function in mice generated after blastomere biopsy, suggesting that the altered epigenetic reprogramming during early embryo development may be the latent mechanism, which results in a hypomethylation status in the brain [77]. Recently, it has been made progress in exploring the effects of embryo culture, culture media, and oxygen tension on epigenetic regulation, although in humans it is difficult (and with ethical implications) to isolate the role of embryo culture on epigenetic perturbations in the developing embryo [105]. In addition, other factors such as temperature, osmolality, pH, and embryo density during culture may potentially impose stress and have significant epigenetic consequences.

Many studies have investigated and are currently investigating the potential risk of ART for the long-life health of children conceived by this technique due to the presence of epigenetic alterations [103,109]. Although concerns about the risk of some diseases have been supported by experimental data, very little evidence has been reported about an increased risk of neurological disorders. Despite early reports on animal models evidencing an increased risk of behavioral, other that metabolic, problems in the offspring generated by in vitro fertilization, the large majority of the studies on the neurodevelopment of children born at full term after ART consistently show that these children are in a comparable condition to normally conceived children, especially when considering more recent surveys [103]. More detailed information will be likely provided by studies reporting longer follow up, especially for children conceived by ICSI. Anyway, at present, alterations in the neurological development do not appear to represent a problem for ART conceived children and should not discourage couples to attend this procedure to get a pregnancy.

## Figures and Tables

**Table 1:** Rodent studies related to neural development derived from cultured embryos.

Author Year [Reference]	Animal Model	Method	Main Findings
Ecker 2004 [75]	129S6/SvEvTac/C57BL/6J F(1) mouse	Behavioral testing	Small but significant long-term alterations in behavior (anxiety, locomotor activity, spatial memory)
Fernandez-Gonzalez 2004 [76]	superovulated female B6CBAF1 mice	developmental and behavior tests	Behavioral alterations in anxiety and displayed deficiencies in implicit memories in mice derived from embryos cultured with FCS Influence of the mRNA expression of multiple growth-related imprinted genes in blastocysts cultured in the presence of FCS
Khosla 2001 [27]	superovulation induced in (C57BL/6J 3 CBA/Ca) F1 females	Gene Expression DNA Methylation	Reduction of weight in M16+FCS fetuses decreased expression of the imprinted *H19* and *IGF2* genes associated with a gain of DNA methylation at an imprinting control region upstream of *H19* increased expression of the imprinted gene *Grb10*.
Wu 2014 [77]	ICR mice	Spatial learning assay pole climbing test MeDIP QRT- PCR Western blotting Microarray	In aged biopsied mice: -Poor spatial learning ability -Increased neuron degeneration -Alteration of proteins expression involved in neural degeneration -Low methylation in the brains

FCS, fetal calf serum; MeDIP, Methylated DNA immunoprecipitation; QRT-PCR, Quantitative real-time PCR.

**Table 2:** Human Studies related to neurological disorders in ART-conceived offspring.

Author Year [Reference]	Study Design	Sample size (n Children)	Main Findings
Stromberg 2002 [79]	population-based retrospective cohort	5680 IVF 11360 NC. 2060 IVF twins 4120 NC	More likely to need habilitation services in IVF children (OR 1.7, 95% CI 1.3–2.2). IVF Children and IVF singletons had an increased risk of cerebral palsy [3.7 (2.0–6.6)] and 2.8 (1.3–5.8) respectively]. IVF twins did not differ from control twins with respect to risk of neurological sequelae.
Lidegaard 2005 [80]	follow-up	6052 IVF 442 349 NC	80% increased risk of cerebral palsy in IVF singletons. Equal frequencies of childhood cancers, mental diseases, congenital syndromes and developmental disturbances
Hvidtjørn 2006 [81]	population-based, cohort	9255 IVF 394713 NC	Increased risk of CP in IVF children
Zhu 2010 [83]	Cohort	3617 IVF/ICSI 3000 OI (with or without IUI), 13462 unplanned pregnancies	Increased risk of CP in IVF/ICS Children
Källén 2010 [84]	Case-control	31.587 IVF 2,623.517 NC	A doubled risk for CP among IVF children
Goldsmith 2018 [85]	Cohort study	203352 NC 1306 IVF 621 ICSI	increased two-fold of CP prevalence in IVF/ICSI mediated mostly by preterm and multiple births similar clinical outcomes between ART and NC children with CP
Punamäki 2016 [89]	Prospective follow-up (7–8-year)	278 NC ART group: 164 IVF, 76 ICSI	Lower levels of cognitive problems in ART boys than in the NC boys Higher levels of cognitive problems in ART girls than in the NC girls IVF/ICSI children did not differ in terms of mental health or developmental outcomes No significant gender differences within the ART group
Maimburg 2007 [91]	Case-control	473 infantile autism 473 controls (33 ART = 10 cases and 23 controls)	Lower risk of developing infantile autism in the ART children
Hvidtjørn 2011 [92]	Population-based follow-up (4–13 years (median 9 years)).	588 967 NC 14 991 IVF 18 148 OI	No ASD risk in ART children
Lehti 2013 [93]	Case-control	4164 autistic cases 16 582 controls In the whole sample: 63 IVF and 229 no IVF	No significant association between IVF and ASDs or its subtypes childhood autism, Asperger’s syndrome or other pervasive developmental disorder
Kissin 2015 [95]	Population-based retrospective cohort	42383 ART (5-year observation period)	The incidence of autism diagnosis remained at 0.8% among singletons, 1.2% among multiples A higher incidence of autism diagnosis in ICSI compared with conventional IVF and a lower when parents had unexplained infertility (among singletons) or tubal factor infertility (among multiples) compared with other types of infertility
Levin 2018 [98]	Population-based cohort	237.863 NC 2.603 IVF 1.721 OI	Attention deficit/hyperactivity disorders and headaches were more common in the OI group, sleep disorders in the IVF group Autism and CP comparable between the groups
Davidovitch 2018 [99]	Cohort	108.548 male offspring	No association between IVF treatment and ASD Association between progesterone hormone treatment and increased risk of ASD

CP, Cerebral Palsy; IUI, intrauterine insemination; OI, ovulation induction; NC, naturally conceived controls; ASDs, autism spectrum disorders.
